# Associations between Positive Health-Related Effects and Soundscapes Perceptual Constructs: A Systematic Review

**DOI:** 10.3390/ijerph15112392

**Published:** 2018-10-29

**Authors:** Francesco Aletta, Tin Oberman, Jian Kang

**Affiliations:** UCL Institute for Environmental Design and Engineering, The Bartlett, University College London (UCL), Central House, 14 Upper Woburn Place, London WC1H 0NN, UK; f.aletta@ucl.ac.uk (F.A.); t.oberman@ucl.ac.uk (T.O.)

**Keywords:** soundscape, environmental noise, public health, well-being, quality of life, restoration

## Abstract

In policy-making and research alike, environmental sounds are often considered only as psychophysical stressors, leading to adverse health effects. The soundscape approach, on the other hand, aims to extend the scope of sound-related research to consider sounds as resources, promoting healthy and supportive environments. The ISO 12913-1 standard defined soundscapes as acoustic environments “as perceived by people, in context.” The aim of this study was assessing associations between positive soundscapes (e.g., pleasant, calm, less annoying) and positive health-related effects (e.g., increased restoration, reduced stress-inducing mechanisms, etc.). Studies collecting data about individual responses to urban acoustic environments, and individual responses on psychophysical well-being were selected, looking at cases where positive effects were observed. The Web of Science, Scopus and PubMed databases were searched for peer-reviewed journal papers published in English between 1 January 1991 and 31 May 2018, with combinations of the keywords “soundscape” and at least one among “health”, “well-being” or “quality of life.” An additional manual search was performed on the reference lists of the retrieved items. Inclusion criteria were: (1) including at least one measure of soundscape dimensions as per the ISO 12913-1 definition; (2) including at least one health-related measure (either physiological or psychological); (3) observing/discussing a “positive” effect of the soundscape on the health-related outcome. The search returned 130 results; after removing duplicates, two authors screened titles and abstracts and selected 19 papers for further analysis. Seven studies were eventually included, with 2783 participants in total. Each study included at least a valence-related soundscape measure. Regarding the health-related measures, four studies included physiological monitoring and the remaining three included self-reported psychological measures. Positive soundscapes were associated with faster stress-recovery processes in laboratory experiments, and better self-reported health conditions in large-scale surveys. Due to the limited number of items and differences in measures across studies, no statistical analysis was performed, and a qualitative approach to data synthesis was sought. Results support the claim that, in contrast with looking at noise only as an environmental stressor, sound perception can act as an enhancer of the human experience in the urban realm, from a health-related point of view.

## 1. Introduction

The adverse health effects of environmental noise on people and communities have been thoroughly investigated by world-wide research institutions and international health organizations over the past decades [1,2,3]. Particularly in cities, excessive exposure to noise has been proved to account for a wide range of psychophysical detrimental health effects, such as: Increased risk of ischaemic heart disease, sleep disturbance, cognitive impairment among children, annoyance, stress-related mental health risks, and tinnitus [4,5]. As this is a substantial public health issue, regulatory bodies, public authorities, and policy makers have put a lot of effort into reducing environmental noise exposures, and noise levels more generally. In the European Union, such efforts are reflected in the “Environmental Noise Directive” (END) [6] and a number of other technical reports and documents issued by EU agencies [7,8], which constitute the references for Member States for the “assessment and management of environmental noise” [6]. The END deals with the management of specific sources; in particular: Road and rail vehicles and infrastructure, aircraft, outdoor and industrial equipment and mobile machinery. As it can be observed, the focus is on typically “unwanted” sound sources and possible nuisance they generate: Little is said in the document about “wanted” sounds, the sounds of “preference”, or the possibility that being exposed to certain acoustic environments can actually induce positive rather than negative moods.

While reducing environmental noise in cities can certainly generate economic and social benefits, in principle it did not always lead straightforwardly to improved well-being and quality of life for people [9,10,11], as sounds might also be important because they transfer information, and loudness can even be desirable in some contexts [12].

Being underpinned by a more holistic approach, soundscape research aims indeed to compensate for this issue, by shifting the research focus from the negative to the positive effects of environmental sounds [13,14,15,16]. The ISO 12913-1 standard defines the term soundscape as “an acoustic environment as perceived or experienced and/or understood by a person or people, in context” [17]. Sometimes “soundscape” is used in community noise literature as an equivalent for “acoustic environment” [16], but the ISO 12913-1 definition highlights that they can be treated as separate concepts: The former relates to the perceptual outcome of the individuals, the latter relates to the whole set of sources generating sounds in a specific space and its physical implications. As such, acoustic environments are neither good nor bad: When the sound sources composing them become “wanted” or “unwanted” for a listener, they are likely to elicit positive or negative soundscapes, accordingly.

The corpus of soundscape studies is growing steadily, and their outreach is increasing accordingly [18], which is promising evidence that the course of this research field is changing. Several studies have tried to provide scientific evidence of the benefits of the “soundscape approach” for public engagement, health, and well-being [19,20,21,22]; however, the lack of empirical and analytical evidence to support that claim is still perceived as a crucial issue by the scientific community [23].

To the best of the authors’ knowledge, no review has been performed so far on the association between the individuals’ positive perception of acoustic environments (i.e., positive soundscapes) and positive health-related effects. Thus, the aim of this paper is to explore such relationships and to identify potential paths to extend the scope of the soundscape approach towards environmental and public health research [23]. For this purpose, we carried out a systematic review of the literature in main scientific databases. The underpinning research question was whether statistically significant associations exist between positive soundscape dimensions and positive health-related effects. It is worth pointing out that, as a perceptual outcome, any soundscape can either be positive or negative. The main theoretical reference of this review to define a positive soundscape is the circumplex model for soundscape characterisation proposed by Axelsson and colleagues [24]. This model has two orthogonal components: The horizontal component is a valence-related dimension (annoying-pleasant), while the vertical component is an activation-related dimension (uneventful-eventful). A soundscape that is both: Pleasant and eventful will be vibrant; pleasant and uneventful will be calm; annoying and uneventful will be monotonous; annoying and eventful will be chaotic. Having this two-dimensional space as reference, any perceptual outcome that can be located in the pleasant region of the model (e.g., pleasant, calm, vibrant, or similar) can be considered as a positive soundscape, while any perceptual outcome that can be located in the annoying region of the model can be considered as a negative soundscape (e.g., annoying, monotonous, chaotic, or similar). Regarding the health-related effects, the definition of “positive” is somewhat more intuitive: It can be assumed that positive health effects occur when, either in a measured or self-reported configuration, enhanced restoration and recovery mechanisms or reduced stress-inducing mechanisms are observed.

## 2. Methods

Given the exploratory nature of this study, there was no pre-defined protocol registration for this review. The basic process and data extraction forms were agreed upon at the beginning of the review work. The study was performed and reported in accordance with the PRISMA (Preferred Reporting Items for Systematic Reviews and Meta-Analyses) guidelines for systematic reviews [25].

### 2.1. Search Strategy and Eligibility Criteria

Studies were selected if they collected data about individual responses to (urban) acoustic environments, and individual responses on psychophysical well-being, looking at positive effects of the former on the latter. The specific inclusion criteria were: (1) Including at least one measure of soundscape dimensions as per the ISO 12913-1 definition [17]; (2) including at least one health-related measure (either physiological or psychological); (3) observing/discussing a “positive” effect of the soundscape on the health-related outcome. Only peer-reviewed journal articles published in English were considered.

The ISO 12913-1 standard highlights that soundscape is a perceptual outcome, deriving from the experience of a physical phenomenon (the acoustic environment) [17]. Thus, any perceptual construct (e.g., calmness, excitement, pleasantness, annoyance, etc.) related to the experience of an acoustic environment could be seen as a potential item on which it is possible to gather individual responses [24,26,27]. These perceptual constructs (soundscape dimensions), when clearly identified and/or formalised, have indeed previously been defined in literature as “soundscape descriptors”, which are “measures of how people perceive the acoustic environment” [10]. Soundscape researchers typically make use of soundscape descriptors associated to scales (e.g., numerical scales, Likert scales, etc.) and ask people to assess their experience of the acoustic environment on those [28,29,30]: Within the framework of this review, soundscape descriptors associated with scales (or other metrics/observations) will therefore be considered as “soundscape measures.”

Regarding the well-being and health-related measures, these were in turn considered to relate to two main groups, namely: (1) Physiological measures: e.g., heart rate, respiratory rate, electrodermal activity or skin conductance level, etc.; (2) psychological measures: e.g., self-reported health condition, satisfaction, quality of life, etc. The rationale for selecting the former group is that most of them are related to the autonomic function (parasympathetic and sympathetic activation) and consequently stress recovery and relaxation or excitement and arousal, which influence health and well-being, and have been reported to be influenced by the acoustic environment [31,32]. The sympathetic system is responsible for stimulating the body’s “fight-or-flight” response, whilst the parasympathetic system is responsible for the stimulation of “rest-and-digest” or “feed and breed” activities, which occur when the body is at rest, or preparing for relaxation. Studies of this kind are typically performed in laboratory settings and include small samples of participants. On the other hand, psychological measures of the latter group are instead more often included within broader (large-scale) socio-acoustic surveys.

Studies were identified by searching Web of Science, Scopus and PubMed electronic databases, by manually scanning the reference lists of retrieved items and through consultation with experts in the field. A combination of the following keywords was used: Soundscape; and at least one among: Health, well-being, or “quality of life”. The search was applied between 1 January 1991 and present. The last search was performed on 31 May 2018. Using two or three databases is a common approach in systematic reviews [25]; the three mentioned above have been shown to be effective in covering most of the relevant soundscape literature [16,18,33].

The assessment about the eligibility of the study was performed independently in a non-blinded standardized manner by two reviewers; a few disagreements between reviewers about inclusion/exclusion of some items were resolved by consensus.

### 2.2. Data Extraction

Information was extracted from each included study on: (1) Soundscape measure(s); (2) health-related measure(s); (3) number of participants of the study; (4) study design; (5) sound levels (or range thereof) and corresponding metrics considered to define exposure conditions; and (6) main observed positive effect(s) of soundscapes on health and well-being.

Considering the unresolvable differences in the metrics across the selected studies, a quality assessment and quantitative meta-analysis under the quality-effects model were not possible [34]. Therefore, a qualitative approach to data synthesis was adopted to answer the review question.

### 2.3. Rationale for the Review

**Figure 1:**
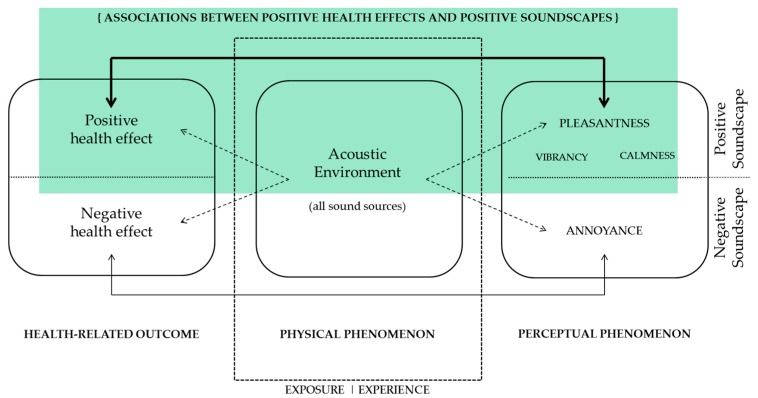
Schematic representation of the theoretical framework underpinning the systematic review: The inclusion criteria were defined to detect studies that established clear associations positive soundscapes and positive health-related effects (area highlighted in green), instead of negative soundscapes and negative health effects (bottom part of the figure), and to dismiss those focusing only on either the perceptual outcome or the health-related outcome (dashed arrows).

The main aim of this review was supporting the claim that the positive perception of acoustic environments might be beneficial for human health and promote more positive living experiences: Both the health effects and the soundscapes can be seen as outcomes of the exposure to a specific acoustic environment. In order to support this hypothesis, it was then necessary to include studies that drew clear associations between these two domains, in the most objective possible way. Figure 1 summarises the general theoretical framework for this review. A considerable amount of literature has looked at this topic only from one side: Either by checking for differences in perceptual outcomes between groups experiencing different acoustic environments, but without considering the health implications on the sample [29,35,36]; or by investigating the health impacts (either supportive or detrimental) of exposure to different acoustic environments on different populations, but dismissing the perceptual constructs being elicited [37,38]. The novelty of this review lays in the fact of looking at associations between health effects and perceptual responses directly (regardless of the acoustic environments inducing them) and focusing on the positive outcomes (instead of detrimental effects) deriving from the interactions between these two. Therefore, among the inclusion criteria of this review, two of them were the most crucial ones: Having considered a health-related measure, and having considered a soundscape measure on the same sample of participants. Those two types of measures were conceived by the reviewers in the broadest possible fashion.

## 3. Results

**Figure 2:**
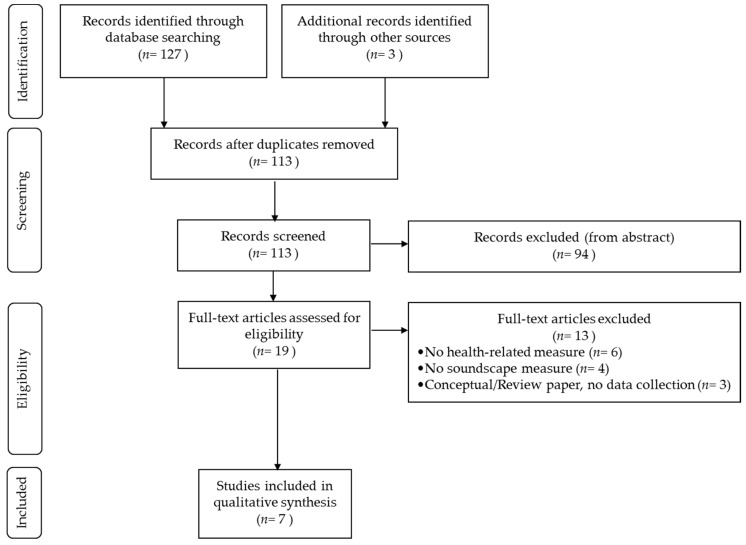
Flow of information through the different phases of the systematic review [25]. The number of studies included in the qualitative synthesis does not equal the difference between the articles assessed and those excluded (*n* = 6), because one of the selected articles included two separate studies, both meeting the eligibility criteria.

The search through the three databases and the additional manual search returned 130 results. After removing duplicates, the abstract of 113 records were read by two authors and 94 items were excluded because the topic of the papers was not relevant (e.g., different research field) and/or did not address the review research question. The full-texts of the remaining 19 papers were accessed and 13 of them were excluded because they failed to meet the eligibility criteria (e.g., lack of either the soundscape or the health-related measure, the paper did not actually include data collection, etc.). The remaining six papers were included in the review; one of those reported on two separate studies, thus seven studies were eventually considered. Figure 2 summarises the selection process of the review records.

Table 1 shows the data extracted from the seven studies considered in the review, reported according to the chronological order of publication. It is important to notice that, due to the differences in study designs, the selected studies used substantially different metrics for the characterisation of the acoustic environments and/or auditory stimuli. Laboratory studies typically referred to equivalent levels (L_eq_) while socio-acoustic surveys made use of daily-averaged levels (e.g., L_day_, L_den_). This makes it difficult to compare sound levels or exposure across studies, but they are reported in any case for descriptive purposes.

Four studies [39,40,41] included soundscape measures that are either standardized or well-established in soundscape literature [24,27,30]; all these studies referred to physiological health-related measures and made use of laboratory experiments, thus using relatively small samples of participants. The other three studies included in the review [42,43,44], on the other hand, referred to annoyance-related soundscape measures [10,45,46]. In this review “annoyance” is also considered a soundscape measure, consistently with the model proposed by Axelsson and colleagues [24]; reduced annoyance can be interpreted as a positive soundscape outcome. These three studies included psychological health-related measures instead and were performed within large-scale environmental surveys (the acoustic aspects of which were not necessarily the main ones); thus, they typically included larger samples of participants than the laboratory studies.

For the sake of reporting and discussion, the studies were grouped according to the abovementioned sample size criterion (large- and small-scale) and their main characteristics; methods and results are described in the two following sub-sections, accordingly.

### 3.1. The Large-Scale Studies

Öhrström, et al. [42] investigated the benefit of access to quietness in reducing the annoyance from road traffic noise through a set of large-scale socio-acoustic surveys about environmental perception at home in Gothenburg and Stockholm. Their study included 956 participants who were asked several groups of questions about (among others): Noise annoyance, general physical health, and mental well-being. The soundscape-related question (i.e., noise annoyance) was evaluated through two standardized annoyance scales [46]: A five-point categorical scale and numeric 0–10 scale (ranging from “not at all annoyed” to “extremely annoyed”). The verbal standardized annoyance question was: “Thinking about the last (12 months or so), when you are at home, how much does noise from road traffic bother, disturb, or annoy you?”. The health-related part of the questionnaire consisted of a number of items, asking how often (“seldom/never”; “a few times a month”; “a few times a week”; or “everyday”) participants experienced/felt: “Very tired”, “headache”, “stressed”, “unsociable and preferred to be alone”, “irritated and angry”, “stomach discomfort”, “worried and nervous”, and “sad and depressed.” The sample was stratified by “access to a quiet side at home” (i.e., low or high exposure to road traffic noise). For the purpose of the review, this variable is not relevant, as it only relates to the authors’ experimental design and transcends its main aim, which is seeking the association between the health items and soundscape annoyance. Indeed, the authors reported that, in any case (regardless of low or high exposure), lower noise annoyance was associated with improved (i.e., less occurring) experience of health-related issues. Statistically significant relationships (*p* < 0.01, Spearman correlation coefficient, *r*_s_) were observed between noise annoyance (soundscape dimension) and heath items: “Very tired”, *r*_s_ = 0.246; “stressed”, *r*_s_ = 0.224; “irritated/angry”, *r*_s_ = 0.216, “headache”, *r*_s_ = 0.197; and “unsociable”, *r*_s_ = 0.191.

Booi and van den Berg [43] conducted another socio-acoustic survey that included 809 participants, within a broader study about quiet areas in Amsterdam. For the soundscape measure the authors developed a “Need for Quietness” construct, which was based on two scales: “How important is quietness to you: (1) In/around home; (2) in the neighbourhood. Answers were provided for each item on a five-point scale ranging from 1 (“not at all important”) to 5 (“very important”). The internal consistency of the two scales for the Need for Quietness perceptual construct was tested through a Cronbach’s α test (0.711). According to the ISO 12913-1 definition, the Need for Quietness can be considered as a soundscape measure, as it is a perceptual outcome emerging from the experience of an acoustic environment. In this context, lower scores of Need for Quietness can be interpreted as a positive soundscape, as it implies lower psychological burden on the subjects. The health-related measure was based on a single question: “How is your health in general?”. The categorical answers were: “Excellent”; “very good”; “good”; “reasonable”; “poor”. The scores for this question were then recoded into a simpler two-level “health” variable (i.e., good; poor/bad). An independent-sample *t*-test revealed statistically significant differences (*p* < 0.05) in terms of Need for Quietness between the good (M = 3.7) and poor/bad (M = 4.0) groups of the health variable. Thus, the authors concluded that, for their sample, a better health condition was associated with greater satisfaction with everyday soundscape experience (i.e., lower need for quietness).

Shepherd et al. [44] carried out a study in New Zealand including 823 participants in total, residing across a different gradient of urbanization (i.e., rural areas of New Zealand’s North Island, areas around Auckland International Airport, and Auckland city). For the health-related dimensions, the surveys referred to the standardized protocol for Health-Related Quality of Life (HRQOL), as a proxy for the influence of the individuals’ health status on their global well-being. The HRQOL was assessed using the reduced version of the World Health Organization quality of life protocol, the WHOQOL-BREF (World Health Organization Quality of Life) [47,48]. This instrument includes two general items on self-reported health and quality of life, and 24 items representing four HRQOL domains; namely: Physical health (seven items), psychological wellbeing (six items), social relationships (three items), and environmental factors (eight items). In this protocol items are rated using a five-point scale, ranging from negative (1) to positive (5) evaluations of HRQOL domains. As a soundscape measure, the dimension of “annoyance” was assessed also in this case. Questions referred to noise from road traffic, neighbours, or other sources and participants could assess them on a five-point scale ranging from “Not annoyed at all” (1) to “Extremely annoyed” (5). The annoyance scores were pooled and dichotomised into a new variable (i.e., “not annoyed” and “most annoyed”) to identify two groups within the sample. The most annoyed group had lower mean scores than the not annoyed group for the HRQOL domains. A set of ANCOVA tests [49] revealed statistically significant differences between the most annoyed and not annoyed groups for all the HRQOL domains: Physical (*F* = 41.799, *p* < 0.001); psychological (*F* = 36.02, *p* < 0.001); social (*F* = 14.984, *p* < 0.001), and environmental (*F* = 64.83, *p* < 0.001). The authors concluded that, while unpleasant soundscapes can induce annoyance, positively evaluated soundscapes can support restoration and quality of life.

### 3.2. The Small-Scale Studies

Alvarsson et al. [39] carried out a laboratory experiment where 40 participants were exposed to four different environmental sounds after a stressful mental arithmetic task to see what sounds would provide a faster recovery from the stressor event. The four sounds were: Nature sound (i.e., tweeting birds mixed with sounds from a fountain—50 dB); high traffic noise (i.e., road traffic noise recorded close to a densely used road—80 dB); low traffic noise (same stimulus as the previous one, set at a lower sound level—50 dB); and ambient noise (i.e., sound from a quiet backyard, mixed with constant and low ventilation noise—40 dB). During the recovery phase of the experimental design, while the auditory stimulus was being provided, the health-related measures were taken. They consisted of the skin conductance level (SCL), which was used as a proxy for sympathetic activation, and high frequency heart rate (HR) variability, which was used as a proxy for parasympathetic activation. In terms of soundscape measures, the participants were asked to assess the four environmental sounds used during the experiment in terms of pleasantness, eventfulness, and familiarity. The authors reported that these descriptors were assessed through bipolar categorical scales, but no further details were provided; thus, it is assumed the question was posed on a scale related to each item, ranging from “not at all” to “completely”, or similar. While HR variability showed no effect, SCL decrease (interpreted as stress recovery) tended to be faster during exposure to natural sound compared to noisy environments. In an ANCOVA test, with baseline (i.e., silence) as covariate, the interaction between sound type and stress recovery time was found to be statistically significant (*F* = 1.34, *p* = 0.034). Likewise, nature sounds were assessed as being the most pleasant and familiar (positive dimensions of soundscape appraisal). These results suggest that positive soundscapes can facilitate recovery from sympathetic activation after a psychological stressor.

A similar laboratory setting was prepared by Hume and Ahtamad [40]. Their experiment included 80 participants. The participants were exposed to eight different auditory stimuli (e.g., kids playing, fire siren, birdsong, etc.) in a randomized sequence. The health-related measures consisted of heart rate (HR), respiration rate (RR) and electromyography (EMG); these were taken immediately before (baseline) and during the participants’ exposure to each stimulus. The soundscape measures were instead taken subsequently to the exposure: Immediately after listening to the audio excerpt, participants had to score the pleasantness and arousal of the stimuli on two nine-point scales ranging from “extremely unpleasant” and “no arousal” (1) to “extremely pleasant” and “extremely aroused” (9), accordingly. The associations between the three health-related measures and soundscape measures were tested separately through linear mixed model ANOVAs. For the health variables, the change (i.e., increase or decrease) of the physiological measures during the sound exposure was considered, rather than absolute values. The pleasantness scores were recoded into a new three-level ordinal variable: Most unpleasant (scores 1–3), neutral (scores 4–6), and most pleasant (scores 7–9). The authors report it was not possible to draw conclusions on the arousal dimension, since the sample of participants failed to achieve a full range of arousal estimates (possibly due to an incomplete understanding of the concept, compared to pleasantness). In terms of heart rate, the more unpleasantly scored sounds resulted in the largest HR decrease, whilst the most pleasant sounds were associated with the smallest HR decrease. These differences were found to be statistically significant (*F* = 2.153, *p* = 0.029). For the respiration rate, a positive relationship was observed: An increased RR was associated with an increase in pleasantness. These differences were statistically significant too (*F* = 107.8, *p* < 0.001). For the electromyography, there was no general separation measured between the baseline and the exposure, as observed instead for the HR and RR results; thus, the authors did not report any statistical test on this last variable. Although no detailed interpretation is provided about the health implications of the observed physiological profiles, the authors generally concluded that soundscapes have the potential to (positively) affect health by observing that for soundscapes assessed as unpleasant, there was a trend for HR to decrease and RR to slightly increase, while for soundscapes assessed as pleasant, there was a minimal HR decrease and a clear RR increase. To some extent, this issue is left unsolved by the authors who did establish the association but did not offer further insights into its causal connections, and more research would be desirable to explain that particular physiological response.

The paper by Medvedev et al. [41] reported on two separate studies, which addressed similar research questions from slightly different perspectives, to support the authors’ research hypothesis. The listening experiments shared the same methodology (i.e., exposure of participants to sound, physiological monitoring, and self-reported soundscape appraisal), but were independent of one another (different samples of participants, different stimuli and procedure, etc.); therefore, for the sake of this review, they were considered as separate items, since Study 1 and Study 2 alike met the inclusion criteria alone. The health-related measures consisted of continuous monitoring of heart rate (HR) and skin conductance level (SCL). The soundscape measures consisted of self-reported appraisals based on seven-point Likert scales ranging from “not at all” to “completely” for the following soundscape dimensions: Pleasantness, arousal, familiarity, eventfulness, and dominance. In Study 1, participants were asked to perform a stressful arithmetic task, and for the recovery phase they were exposed to four different environmental sounds (i.e., birdsong, ocean, road noise, and construction). On the other hand, in Study 2, there were six slightly different stimuli (i.e., aviation, motorcycle, construction, ocean, birdsong, classical music) and the scenarios started “at rest” (no cognitive task): Participants were asked to listen passively to the stimuli and to assess the soundscape after each scenario. The same health and soundscape measures were taken as in Study 1. The authors performed a set of paired comparison tests to explore the effects of soundscapes on stress recovery (i.e., decrease of SCL). A statistically significantly faster decrease of SCL (*p* < 0.01) was observed for soundscapes rated as the most pleasant and the least eventful, compared to the least pleasant and most eventful. Furthermore, a statistically significant difference (*p* < 0.05) was observed between sounds assessed as most and least familiar. Study 2 looked at the same issue, from a “symmetric” perspective. Paired comparison tests showed that soundscapes assessed as the least pleasant and familiar, and the most dominant, were associated with significantly larger (*p* < 0.05) increases of the SCL, compared to the soundscapes ranked as the most pleasant and familiar, and the least dominant, accordingly. By integrating the results from the two studies, the authors concluded that the experience of pleasant soundscapes would facilitate faster recovery from stress, compared to unpleasant soundscapes.

## 4. Discussion

### 4.1. Differences in the Methodological Backgrounds of the Reviewed Studies

The review identified studies that could approximately be sorted into two categories: The large-scale studies (see Section 3.1) and the small-scale studies (see Section 3.2); the two categories were underpinned by substantially different methodological approaches. Studies from the former group tend to be led by annoyance-related analyses. It should be noted that, being a valence measure and a perceptual construct itself, noise annoyance can be considered a soundscape dimension in its own right (thus, meeting the inclusion criteria). However, such studies are possibly inspired by different principles. In general, they aim at estimating long-term effects (e.g., “thinking about the last 12 months…” and similar questions), focus on specific contexts (e.g., experience of sound/noise at home or at work), and consequently need large samples of participants to achieve reasonable robustness. On the other hand, studies from the latter group were found to be more soundscape-oriented. They are the outcomes of projects that typically had the perceptual domain as a driving research component. They are more aligned to the soundscape approach of contextual experience (i.e., “right here, right now”) and are more focused on the immediate response of participants to auditory stimulation, thus mainly disregarding long-term effects.

Globally, the number of soundscapes studies is increasing with time [18]. Although, interestingly enough, the type of studies one usually retrieves in “classical” soundscape literature did not emerge within the context of this systematic review [16]. Indeed, the vast majority of soundscape studies nowadays tend to refer to data collection on individual responses about the acoustic environments experienced by people on site, with a relatively limited set of methods, such as: Soundwalks, questionnaires/interviews, non-participant behavioural observations, etc. [10,13,50,51]. These approaches encounter growing consensus in the soundscape research community, because of the ecological validity provided by their results, which is often compromised in laboratory settings [52], or unattended large-scale socio-acoustic surveys. Notwithstanding, none of such studies was detected by the review criteria, as they failed to include any health-related measure. A future direction for research that is possibly worth exploring, therefore, could be the inclusion of additional items related to health, well-being and quality of life, in the protocols of soundscape studies. Currently, standardized questionnaires do not consider this aspect [26,27], even if a number of reduced health-related protocols have already been validated in public health research and proved to properly relate also to psychometric properties in sound-related research [48,53,54]. This would offer further materials and analysis to establish stronger associations between soundscapes experience and positive effects on human health.

### 4.2. Key Results

The results from the items of the first group (large-scale studies) all show a consistent trend; that is: Better self-reported health-related conditions were always associated with reduced noise annoyance. In the specific case of Booi and van den Berg [43], the less urging need for quietness was interpreted as being expression of a reduced noise annoyance (i.e., no need for remediation means). In all studies, the associations were statistically significant with at least a *p* < 0.05 significance level.

Results from the items of the second group (small-scale studies) also show positive effects of experiencing good soundscapes on well-being. The health measures in these studies referred to the autonomic function; i.e., sympathetic or parasympathetic activation. In general, the studies looked at the parasympathetic activation, from a restoration perspective, observing that pleasant soundscapes are associated with more effective stress-recovery, and all those relationships were found to be statistically significant with at least a *p* < 0.05 significance level. The study by Hume and Ahtamad [40] did not provide a detailed interpretation about the relationship between the physiological measures and the soundscape measures, but it specified that the influence of context should be further investigated by considering “contextually supportive” sounds for health.

Taken together, the outcomes of this review support the claim that, both for short-term and long-term (as well as small-scale and large-scale) perspectives, positive perceptions of acoustic environments (soundscapes) can be associated to positive health effects.

### 4.3. Limitations

Due to the substantial differences in measures across studies, it was not possible to perform any statistical or quantitative meta-analysis, and a qualitative approach to data synthesis had to be sought instead. However, this is a common approach in noise-related public health research [34].

Furthermore, the relatively small number of items retrieved might raise some concerns. Notwithstanding, the choice to exclude studies that only collected soundscape data (but did not consider health-related measures) and studies that only assessed health effects of exposure/experience for different sound environments (but did not investigate the actual “perception” of those) was justified in Section 2.3. For both sub-groups, small-scale and large-scale studies, it was not always possible to “quantify” and compare the positive effects in the association between soundscapes and health measures. This was either because of a reporting bias (no means reported, only statistical significance) or because of substantial differences in the experimental designs of the studies (e.g., within-group or between-group designs, etc.).

Apart from that, the two sub-groups identified by the review had then their own characteristics related to the methodological approach. For the large-scale studies, it is almost never reported how the questionnaires were submitted to the participants; for instance: Whether the interview was attended/unattended by the researchers, the forms sent/returned by post, etc. This has the potential to introduce a bias in such a way that it is not possible to gather how participants were instructed and/or what was their understanding of the study. Furthermore, in this case, it is hard to control for objective exposure (noise levels), as metrics vary greatly across studies. However, all studies deal with sound levels that ranged between 30 and 80 dB; that is: Excluding cases of extremely low or high exposures.

The small-scale studies have the limitation of having a very specific scope, as they looked at the soundscape efficiency in supporting stress-recovery mechanisms (e.g., relaxing or calming down). There is indeed ongoing research aimed at reviewing the positive effects of specific sounds (e.g., natural sounds) on health and restoration [55]. However, some other sound sources (e.g., human sounds) could also be considered, as well as the soundscape’s potential to elicit the positive perceptual constructs of excitement, liveliness and, more generally, engaging behaviours [12].

Overall, it is important to acknowledge that, especially for self-reported measures, the association between perception and health related to sound is a complex issue in terms of potential confounding factors, such as the sound level itself (which was not possible to control for in this review, due to the substantial differences of metrics between studies) or other personal elements (e.g., expectation, preconception, familiarity, etc.). Studies dealing with physiological measures are of course less vulnerable to this type of bias. However, more approaches are becoming relevant in soundscape studies to try to overcome the “experimenter bias” and self-reported data, by relying on behavioural observations and covert methods for data collection [50,51].

## 5. Conclusions

This paper reported on the positive health-related effects of experiencing positive soundscapes. For this purpose, a systematic review in accordance with the PRISMA guidelines was performed on three major scientific databases. After the screening process, the dataset resulted in seven items that were sorted into two groups, depending on the scale (i.e., large or small) of the investigation. Having substantially different methodological approaches, the studies were qualitatively analysed. The review pointed out that, regardless of the scale, statistically significant associations exist between positive soundscape perceptual constructs and health benefits. The main conclusions are:

- For the large-scale studies, positively assessed soundscapes (e.g., reduced noise annoyance) are statistically significantly associated with better self-reported health conditions.
- For the small-scale studies, positively assessed soundscapes (e.g., pleasant, calm) are statistically significantly associated with faster recovery from environmental stressors.

The review qualitatively showed that a trend is clearly observable, and more research efforts should be deployed in that direction to support the application of the soundscape approach at a planning and design level [56,57]. The ongoing standardization in soundscape studies [58] will hopefully help better position the discipline in the broader context of environmental research and public health.

## Figures and Tables

**Table 1:** List of studies included in the systematic review in chronological order of publication. The main soundscape and health-related measures, the study design, the sound levels, the number of participants and main conclusions of the studies are reported. Since the studies often included several experimental conditions and sound levels, these are reported as levels range (e.g., 40–80 dB, might include several experimental conditions of 40, 50 and 80 dB: For more specific information it is possible to refer to the original studies).

Reference	Soundscape Measure	Health-Related Measure	Study Design	Sound Levels (Metric)	Participants	Main Conclusion(s)—Observed Positive Effect(s)
Alvarsson et al. [39]	Pleasantness, Eventfulness, Familiarity	Autonomic function (HR, SCL)	Laboratory experiment	40–80 dB (L_Aeq-4min_)	40	Nature sounds facilitate recovery after a psychological stressor.
Booi and van den Berg [43]	Need for Quietness	Self-reported health condition	Socio-acoustic survey	30–75 dBA (L_day_)	809	People with good health have a lower need for quietness.
Medvedev et al. ^1^ [41]	Pleasantness, Eventfulness, Familiarity, Arousal, Dominance	Autonomic function (HR, SCL)	Laboratory experiment	64 dB (SPL)	45	Pleasant soundscapes facilitate faster recovery from stress compared to unpleasant soundscapes.
Medvedev et al. ^1^ [41]	Pleasantness, Eventfulness, Familiarity, Arousal, Dominance	Autonomic function (HR, SCL)	Laboratory experiment	64 dB (SPL)	30	Experience of unpleasant soundscapes at rest produces greater stress than pleasant soundscapes.
Öhrström et al. [42]	Noise Annoyance	Self-reported health and well-being condition	Socio-acoustic survey	35–65 dB (L_Aeq-24h_)	956	Experience of quietness supports health and results in a lower degree of annoyance, disturbed relaxation and sleep, and contributes to physiological and psychological well-being.
Shepherd et al. [44]	Noise Annoyance	Self-reported health and well-being condition	Socio-acoustic survey	55–76 dBA (L_den_)	823	Quiet soundscapes facilitate restoration, and/or impede insult to health.
Hume and Ahtamad [40]	Pleasantness, Arousal	Autonomic function (HR, RR, EMG)	Laboratory experiment	60–74 dBA (SPL)	80	The more pleasant the soundscape, the greater the increase in respiratory rate and the smaller the decrease in heart rate.

^1^ Reference [41] included two separate studies and they are treated as separate items within the current review.
